# Role of Mediterranean diet in endocrine diseases: a joint overview by the endocrinologist and the nutritionist

**DOI:** 10.1007/s40618-023-02169-2

**Published:** 2023-09-11

**Authors:** L. Barrea, L. Verde, G. Annunziata, E. Camajani, M. Caprio, A. S. Sojat, L. V. Marina, V. Guarnotta, A. Colao, G. Muscogiuri

**Affiliations:** 1 Dipartimento di Scienze Umanistiche, Università Telematica Pegaso, Via Porzio, Centro Direzionale, Isola F2, 80143 Naples, Italy; 2 Centro Italiano per la cura e il Benessere del paziente con Obesità (C.I.B.O), Dipartimento di Medicina Clinica e Chirurgia, Unità di Endocrinologia, Diabetologia e Andrologia, Università degli Studi di Napoli Federico II, Via Sergio Pansini 5, 80131 Naples, Italy; 3 Department of Public Health, University of Naples Federico II, Naples, Italy; 4 Department of Pharmacy, Federico II University, 80131 Naples, Italy; 5 Department of Human Sciences and Promotion of the Quality of Life, San Raffaele Open University, 00166 Rome, Italy; 6 Laboratory of Cardiovascular Endocrinology, San Raffaele Research Institute, IRCCS San Raffaele Roma, Via di Val Cannuta, 247, 00166 Rome, Italy; 7 National Centre for Infertility and Endocrinology of Gender, Clinic for Endocrinology Diabetes and Metabolic Diseases, University Clinical Centre of Serbia, Belgrade, Serbia; 8 Section of Endocrinology, Department of Health Promotion, Mother and Child Care, Internal Medicine and Medical Specialties “G. D’Alessandro” (PROMISE), University of Palermo, Piazza delle Cliniche 2, 90127 Palermo, Italy; 9 Dipartimento di Medicina Clinica e Chirurgia, Unità di Endocrinologia, Diabetologia e Andrologia, Università degli Studi di Napoli Federico II, Via Sergio Pansini 5, 80131 Naples, Italy; 10 Cattedra Unesco “Educazione alla salute e allo sviluppo sostenibile”, University Federico II, Naples, Italy

**Keywords:** Mediterranean diet, Endocrine disorders, Thyroid, Gonadal disorders, PCOS, Neuroendocrine tumors, Nutrition, Diet

## Abstract

**Purpose:**

The purpose of this review is to examine the current evidence on the potential role of Mediterranean diet (MD) in the prevention and management of endocrine disorders and to highlight the importance of interdisciplinary collaboration between endocrinologists and nutritionists.

**Methods:**

A literature search was conducted using PubMed and Google Scholar databases to identify relevant studies published in English. Studies were selected based on their relevance to the role of MD in the prevention and management of endocrine disorders. The search terms included "Mediterranean diet," "endocrine disorders," "thyroid disorders," "gonadal disorders," and "neuroendocrine tumors".

**Results:**

The studies reviewed suggest that MD may have a beneficial effect in the prevention and management of various endocrine disorders, including thyroid disorders, gonadal disorders, and neuroendocrine tumors. MD has been associated with decreased risk of nodular thyroid disease and thyroid cancer, improved male and female reproductive health, and a potential role in the management of neuroendocrine tumors. MD's anti-inflammatory and antioxidant properties, as well as its high levels of phytochemicals, may play a role in its beneficial effects.

**Conclusion:**

Interdisciplinary collaboration between endocrinologists and nutritionists is essential for the optimal management of endocrine disorders, including the potential role of MD in their prevention and management. While further research is needed, the current evidence suggests that MD may have a protective effect against endocrine disorders, and its incorporation into dietary recommendations may be beneficial.

## Introduction

Mediterranean diet (MD) is a dietary pattern characterized by high consumption of fruits, vegetables, legumes, whole grains, fish, and olive oil, and low consumption of red meat, processed foods, and simple sugar [1]. Actually, MD is recognized by United Nations Educational, Scientific and Cultural Organization (UNESCO) as an intangible cultural heritage of humanity, and it was defined as a social traditional practice ranging from the landscape to the cuisine of seven Mediterranean countries [2]. This dietary pattern has been associated with numerous health benefits, including a reduced risk of chronic diseases, such as type 2 diabetes mellitus (T2DM), cardiovascular disease, and certain types of cancer [3, 4].

Endocrine diseases are a group of disorders that affect the endocrine system, responsible for hormone production and regulation [5]. These disorders can have a significant impact on overall health and quality of life. Thyroid, gonads, and neuroendocrine systems are all important components of the endocrine system that play a crucial role in maintaining overall health [5]. Thyroid disorders such as thyroid nodules can affect metabolism, energy levels, and mood [6]. Disorders of the male gonadal system, such as hypogonadism, can lead to decreased libido, muscle mass, and bone density [7]. Similarly, disorders of the female gonadal system, such as polycystic ovary syndrome (PCOS) and menopause, can lead to hormonal imbalances, hirsutism and fertility issues [8]. Finally, neuroendocrine tumors (NETs) are a rare type of cancer that can affect various parts of the organism, including the pancreas, lungs, and gastrointestinal tract [9].

Of note, inflammation and oxidative stress play an important role in the pathogenesis and/or progression of various endocrine disorders, including thyroid disorders, gonadal disorders, and neuroendocrine tumors [10]. Chronic inflammation and high levels of reactive oxygen species (ROS) can lead to tissue damage and altered function, contributing to the development and progression of these disorders [10].

Research has shown that MD may have a beneficial effect for various endocrine disorders [9, 11–14]. For example, studies have suggested that higher adherence to MD could help to lower the risk of thyroid disorders, including autoimmune thyroiditis [15, 16]. Additionally, MD has been associated with improved male and female reproductive health [13, 14], including increased sperm quality [13] and improvement of the clinical picture of PCOS [11]. Studies have also suggested that MD may have a beneficial role in NETs [9].

Overall, MD appears to have a protective effect against endocrine disorders through a variety of mechanisms, including its anti-inflammatory and antioxidant properties [17, 18]. In fact, MD provides high levels of phytochemicals, including dietary polyphenols and flavonoids, which have been reported to exert beneficial biological effects, including antioxidant, anti-inflammatory, immunomodulatory, antitumoral, antidiabetic and anti-obesity activities [17]. In this regard, data from the INTERCATH study, an observational study of 1121 patients undergoing coronary angiography found that high sensitive C-reactive protein (hs-CRP), a marker of inflammation that has been linked to an increased risk of cardiovascular disease and other chronic conditions, correlated significantly with adherence to MD [19].

In this context, is clear that interdisciplinary collaboration between endocrinologists and nutritionists is essential for optimizing patient care and improving outcomes in the management of endocrine disorders.

The aim of this review was to provide an overview of the current evidence on the role of MD in various endocrine systems and highlights the importance of interdisciplinary collaboration in managing these conditions.

## Endocrine axes and the Mediterranean diet

### Thyroid axis and Mediterranean diet

#### Point of view of the endocrinologist

The thyroid gland is a crucial endocrine organ that regulates metabolic functions, male and female fertility, growth, and development [20, 21]. Thyroid diseases, including cancers, autoimmune diseases and thyroid dysfunctions, are becoming a serious social problem with rapidly increasing prevalence [22]. The most significant nutritional risk factor for developing thyroid nodular diseases is iodine deficiency [23, 24]. Additionally, iodine intake is associated with thyroid cancer risk [25], and there is a U-shaped relationship between iodine consumption and thyroid diseases, meaning that both low and high intake can lead to thyroid diseases [26]. Various factors, both modifying and non-modifying, could contribute to the increased incidence of benign/malign thyroid nodular diseases [27]. Unhealthy dietary patterns may play a role in the development of thyroid nodular disease, although it is unclear whether this is due to the direct effect of unhealthy nutrition or mediated by factors such as obesity, insulin resistance, and inflammation that often arise from unhealthy dietary habits [23]. Indeed, the thyroid gland is sensitive to inflammation and oxidative stress, which can impair its function and contribute to benign/malignant thyroid nodular diseases [28].

Both inflammation and oxidative stress can contribute to the formation of thyroid nodules [29]. Nodules are abnormal growths in the thyroid gland that can be benign or malignant. Chronic inflammation can lead to the formation of nodules by promoting the proliferation of thyroid cells and disrupting their normal function [30]. A clinical study involving 2722 subjects showed that individuals with high inflammation (assessed through the levels of white blood cell, neutrophil, lymphocyte and monocyte in subjects without obvious infection) had a higher prevalence of thyroid nodules and thyroid-stimulating hormone than those with low inflammation, even after adjusting for metabolic parameters and other confounders [31]. In particular, inflammation can inhibit the synthesis of thyroid hormone by affecting the conversion of thyroxine (T4) to triiodothyronine (T3) in peripheral tissues [32]. Cytokines, such as interleukin (IL)-1, IL-6, and tumor necrosis factor-alpha (TNF-α), can decrease the activity of 5′-deiodinase, which is responsible for the conversion. This results in a decrease in T3 levels and an increase in thyroid-stimulating hormone (TSH) levels as a compensatory mechanism. Cytokines can also decrease the expression of thyroglobulin, which further contributes to the decrease in thyroid hormone levels [32]. It is known that chronic inflammation may be also the result of a persistent immune response. For instance, Hashimoto's thyroiditis, an autoimmune disorder that causes chronic inflammation of the thyroid gland, is a common cause of nodules in the thyroid [33]. Oxidative stress can also contribute to the formation of nodules by promoting genetic mutations and DNA damage that lead to abnormal cell growth [30].

Thyroid cancer is a complex disease that can arise from various genetic and environmental factors [22]. Inflammation and oxidative stress are thought to play a role in its development [30]. Chronic inflammation can promote the growth and survival of cancer cells by providing them with a favorable microenvironment. For example, immune cells and cytokines can promote angiogenesis, the formation of new blood vessels, which is essential for the growth and spread of cancer cells. Inflammatory cytokines can also activate signaling pathways that promote cell proliferation and survival. Oxidative stress can contribute to the development of thyroid cancer by promoting genetic mutations and DNA damage that lead to the transformation of normal thyroid cells into cancer cells [30].

Several studies have investigated the relationship between inflammation, oxidative stress, and thyroid cancer [34–36]. In particular, chronic inflammation is linked to oxidative stress, which can cause DNA damage and thus contribute to the accumulation of cancer-initiating genetic alterations in cells [35]. For instance, a recent study found out that the serum levels of malondialdehyde, a well-established marker for screening and monitoring the oxidation stress level, were significantly higher in 55 patients with differentiated thyroid cancer compared to the healthy subjects [34]. Another study investigated the levels of ROS in 50 malignant and benign thyroid lesions and 41 normal tissues and correlated them with the thyroid differentiation score and the clinic-pathologic features [36]. In both malignant and benign thyroid tissue, the production of ROS and expression of the NADPH oxidase 4 (NOX4), a significant source of ROS, playing an important role in tumor cell proliferation and apoptosis, were found to be higher than in healthy tissues [36]. Follicular thyroid cancers (FTCs) and anaplastic/poorly differentiated cancers exhibited greater oxidative stress compared to papillary thyroid tumors (PTCs) [36]. Furthermore, oxidative stress was higher in FTCs than in follicular adenomas and mutated PTCs showed higher levels of oxidative stress than non-mutated PTCs. In malignant tumors, oxidative stress was negatively correlated with thyroid differentiation score and positively correlated with tumor stage and American Thyroid Association risk [36]. Overall, these findings suggested that thyroid tumors were subjected to increased oxidative stress compared to healthy tissues and that oxidative stress is associated with tumor aggressiveness and mutations in the MEK-ERK pathway in PTC [36].

In more detail, inflammatory mechanisms can contribute to thyroid alterations through the action of immune cells and cytokines [37]. Chronic inflammation can lead to the infiltration of immune cells, such as lymphocytes and macrophages, into the thyroid gland. These cells produce cytokines that can damage thyroid cells and alter their function. For example, pro-inflammatory cytokines like IL-1 and TNF-α can inhibit thyroid hormone synthesis and secretion, leading to hypothyroidism. Conversely, anti-inflammatory cytokines like IL-10 and transforming growth factor (TGF)-β can have a protective effect on thyroid cells and promote their survival [37]. Oxidative stress is another mechanism that can lead to thyroid alterations [32]. It occurs when there is an imbalance between the production of ROS and the body's ability to neutralize them with antioxidants. ROS can damage thyroid cells and contribute to the formation of nodules and thyroid cancer [32]. For example, hydrogen peroxide (H_2_O_2_) can inhibit thyroid hormone synthesis and secretion by damaging thyroid peroxidase, a key enzyme involved in the synthesis of thyroid hormones [38]. ROS can also cause DNA damage and promote genetic mutations that lead to the formation of nodules and thyroid cancer [32].

In conclusion, inflammation and oxidative stress are important mechanisms that can lead to thyroid alterations, the formation of nodules, and thyroid cancer. Chronic inflammation can promote thyroid cell damage, proliferation, and abnormal function, while oxidative stress can contribute to genetic mutations and DNA damage that lead to abnormal cell growth. Better understanding of the mechanisms underlying thyroid alterations and cancer can lead to the development of new diagnostic and therapeutic strategies to improve patient outcomes.

#### Point of view of the nutritionist

There is some evidence to suggest that the anti-inflammatory and antioxidant mechanisms of MD may be helpful in promoting thyroid health and reducing the risk of thyroid nodules and cancer [15, 16]. The formation of thyroid nodules is influenced by various factors, including genetics, iodine intake, and inflammation. The latter, together with oxidative stress, is a key driver of thyroid diseases.

First, since iodine intake, both high and low, is the main nutritional risk factor for nodular thyroid disease [39], MD provides a moderate amount of iodine, which can help promote optimal thyroid function without contributing to nodule formation. It is found in MD through the balanced consumption of whole grains, seafood, lean beef, poultry, milk and dairy products. In this regard, a cross-sectional observational study in 17.197 participants, aimed to analyze the nutrient intake adequacy of MD and Western diet patterns, found that a higher quintile of adherence to MD was associated with a lower prevalence of inadequacy for the intake of several vitamins and minerals, including iodine [40]. The quintiles of adherence to Western dietary pattern showed the opposite tendency. The highest quintile was associated with the highest percentage of individuals with noncompliance of recommendations for the iodine intake [40].

Second, MD is particularly rich in anti-inflammatory (such as polyphenols) and antioxidants compounds (such vitamin C and E) which are found in its variety of vegetable products like fruits, vegetables, legumes, and nuts. For fruits, three case–control studies exhibited a negative correlation between fruits intake and thyroid cancer risk and the authors of the various studies agreed that the cause of this association was due to their high levels of antioxidants, polyphenols, and fiber [41–43]. For vegetables, a metanalysis [44] of 19 case–control studies and a pooled analysis [45] of 11 case–control studies discovered a link between low vegetable consumption and the risk of thyroid cancer, showing a weak negative connection with the intake of all vegetables except cruciferous vegetables. Cruciferous vegetables that include broccoli, cauliflower, kale, cabbage, brussels sprouts and others, contain goitrogens that can block iodine uptake by the thyroid and have been shown to promote thyroid carcinogenesis in rats [46]; however, results from epidemiologic studies on the relationship between cruciferous vegetables and thyroid cancer risk have been inconsistent [47]. Of note, flavonoids are plants pigments with interesting pharmacological properties, such as antioxidant and anti-inflammatory [48]. They are widely present in the plant kingdom and include anthocyanins, flavones, and other pigments [48]. Recent studies conducted in vitro have shown that some flavonoids can be beneficial for thyroid cancer by reducing cell proliferation and increasing cell death [49, 50]. Flavonoids have been found to have effects on anti-proliferation and cell re-differentiation [51]. Specifically, apigenin and luteolin are the most powerful inhibitors of human thyroid carcinoma (papillary, follicular, and anaplastic carcinoma) cell lines in vitro, and also induce the re-expression of sodium iodide symporter (NIS) mRNA in anaplastic thyroid carcinoma cell lines [51]. Additionally, the regular administration of flavonoids for 5 days seems to increase not only the thyroid radioiodine uptake but also the NIS protein and mRNA levels in animal thyroids [52]. Despite the attention paid to the relationship between diet and thyroid cancer risk, it can be speculated that the beneficial anti-inflammatory and antioxidant effects of MD can be also extended to the complex pathogenesis of thyroid nodular disease.

However, it is well established that using a single food as the basis for study does not take into account the synergistic and/or antagonistic interactions that exist between nutrients and foods. Consequently, this approach may not have optimal statistical power to assess associations with thyroid diseases. In this regard, a recent study was conducted to examine the link between adherence to MD as a complete nutritional model and the incidence of thyroid nodular disease in 794 adults (aged 18–65 years; BMI 19.4–55.3 kg/m^2^) [15]. This study had also a second objective of assessing adherence to MD based on the cytological classification of thyroid nodules in the subgroup of study participants undergoing fine-needle aspiration. Authors found that the lowest adherence to MD was significantly associated with the presence of thyroid nodular disease, and in particular, with nodules at high-risk of malignancy. The study also found that patients with TIR5 (the highest risk category) had the lowest adherence to MD compared to other categories. In conclusion, adherence to MD was found to be associated with the presence of thyroid nodular disease and in particular with those at high-risk of malignancy [15].

Currently, the availability of randomized controlled trials (RCTs) specifically examining the effect of MD on thyroid disease is limited. Most research focuses on the general role of adherence to MD in influencing thyroid health but does not specifically examine a MD intervention in comparison with other nutritional approaches. However, observational studies suggest that MD may have benefits for thyroid health due to its nutrient and antioxidant content.

However, more research is needed to fully understand the relationship between diet and thyroid health.

### Male gonadal axis and Mediterranean diet

#### Point of view of the endocrinologist

Infertility has emerged as a significant public health problem, now affecting around 15% of all couples trying to conceive [53]. Male factors attribute to this number with around 30%, while in 20% of couples there is a combination of male and female factor [54]. It is often mentioned in the media that the average male fertility has dropped significantly in the last century. Indeed, a meta-analysis performed in more than 40.000 men has clearly shown a deterioration in semen quality and almost 60% reduction in sperm count in the last 40 years in developed countries [55]. Furthermore, the span of relevant factors influencing male fertility is widening in recent years and now includes a multitude of genetic and lifestyle aspects. The influence of dietary habits becomes important from the very early stages of life, however with a less clear association between pubertal timing and obesity. Some studies show that peri-pubertal boys with overweight and obesity have a lower testicular volume than their age matched controls, which was predictive for a worse sperm production [56], while others have different results, with testicular enlargement occurring significantly earlier in boys with obesity in comparison to a normal-weight based reference cohort [57]. Whichever it may be, maintaining a healthy diet without significant metabolic alterations in childhood and adolescence is very significant to preserve male fertility and escape from the vicious cycle of obesity and male hypogonadism [58]. Apart from age, endocrine disorders and genetic conditions, environmental factors and personal behaviors could be seen as relevant contributors to these results [59–63]. Obesity, sedentary lifestyle, smoking, recreational drugs and alcohol intake, psychological stress, tight clothes, and xenobiotics (pesticides, toxins, pollutants, etc.) all create new challenges in male infertility treatment [59–63]. Novel analysis such as sperm DNA fragmentation, still not routinely assessed in fertility workup, improve fertility and in-vitro-fertilization (IVF) outcomes, and shed light on relevant mechanisms [64]. In males with no predisposing genetic factors, the aforementioned lifestyle factors are significantly associated with the generation of oxidative stress, regarded widely as one of the most significant contributors to deterioration of fertility [65]. A diet rich in simple carbohydrates, animal proteins, saturated and trans fats and poor in fiber, along with obesity is frequently observed in sub fertile and infertile males [66, 67]. High intake of red and processed meat, sugary drinks and alcohol can also lead to increased oxidative stress, which is reported in 30–80% of male infertility cases [68]. As ROS overcome the antioxidant barrier of the spermatozoa, they tend to deteriorate sperm morphology parameters [69], damage the cell membrane lipids, proteins and cell DNA and reduce motility, live sperm count, sperm concertation and oocyte connecting ability [70]. Spermatozoa are also exposed to oxidative stress during their passing in the seminal fluid and epididymis, and the degree of fragmentation is somewhat repairable in the oocyte but a pronounced DNA damage may not be, thus significantly influencing pregnancy outcomes and the fertilization process itself [71]. This in turn can lead to a vicious cycle, since continuous inadequate food choices keep increasing the oxidative stress while hyperinsulinemia and hyperglycemia also influence the hypothalamus-pituitary–gonadal (HPG) axis in separate mechanisms [62]. Excess weight can lead to hypogonadism, reduced testosterone levels and a reduced spermatozoa count [72]. The increased aromatase expression can convert testosterone to estrogen in turn decreasing follicle-stimulating hormone (FSH) and luteinizing hormone (LH) and disrupting the HPG axis further while low testosterone levels add to oxidative stress and mitochondrial disfunction in the Leydig cells [73]. Insulin resistance and impaired glucose tolerance also act in a multitude of ways, trough reducing sperm metabolism and glucose uptake and impairing their function and capacity [74]. It is known that patients with T2DM have an increased sperm DNA fragmentation and lower progressive sperm motility than non-diabetic patients along with a higher partner miscarriage rate [74]. Leptin is another relevant factor in this metabolic cascade, since, produced in excess by the adipose tissue in subjects with overweight/obesity, it affects FSH and LH pituitary release and pulsatility while also maintaining low grade inflammation, increasing ROS and decreasing sperm quality [75]. White adipose tissue is a source of production of ROS and low-grade inflammation mediated by ghrelin, IL-6 and TNF-α contributing to HPG axis disturbances [76]. A multitude of lipid profile alterations in dyslipidemia also contribute to infertility through several aforementioned mechanisms [77]. DNA fragmentation has been shown to be associated with obesity and is a good indicator of a poor spermatogenesis quality [78]. All this data shows us that diet has a major role in male infertility treatment, and a good dietary balance with plenty of foods containing antioxidants and antioxidant supplementation together with weight loss is always recommended [79].

MD embodies certain healthy food choices and food preparation techniques that significantly improve the aforementioned negative consequences of an inadequate diet, obesity and metabolic disbalances. The data on positive effects of MD on male reproductive health are not in abundance, but the existing research is mostly in line with other health benefits, since significant improvements in sperm quality, count and improved conception rates were shown [80]. A balanced diet rich in vegetables, fruit and whole grain food increases sperm count and motility [81, 82] and their bioactive compounds act via sperm mitochondria reducing oxidative stress and preserving optimal mitochondrial function [83, 84]. Resveratrol, for example, found in grapes, was shown to have an antioxidant and an anti-inflammatory effect [85]. Polyunsaturated fatty acids (PUFA) n-3 aid in metabolic function of the sperm cells while the addition of olive oil can have a positive effect in reducing the damage from oxidative stress and add to the sperm membrane lipid layer, thus improving the sperm quality [13]. Vitamins and other compounds found in these foods further reduce oxidative stress. The effect of MD on assisted reproductive technologies (ART) outcomes is also a point of discussion, however, there is a lot of variables and contributing factors influencing these results and further studies are necessary in this field. Even though the existing evidence do not support the theory that nutritional choices and food are a relevant factor for testicular cancer [86], several studies have shown certain associations between these types of tumors and dietary fat, cheese and even fiber intake, however, with very conflicting results [87].

All in all, due to the growing amount of research done in this field with various dietary choices and lifestyle alterations in relation to metabolic health, hormonal axis and male and female infertility, certain new compounds and novel dietary choices can emerge as relevant both in a positive and negative context. However, it is always necessary that this data is put in a broader perspective, since a well-balanced diet, with proper food choices, regular meals and adequate physical exercise remains a staple of metabolic, cardiovascular, reproductive, and overall health.

#### Point of view of the nutritionist

Diet exerts a relevant impact on the gonadal axis, in general, and, more specifically, on male fertility [88]. Such impact can be positive or negative, depending on dietary habits [13]. Besides consuming (or avoiding) specific foods or supplements, research focused on the effects of the global diet pattern, identifying MD as the best one. In this sense, MD is recognized for its health-promoting potential [89], as well as for its beneficial impact on male fertility [13]. This has been reported in various cross-sectional studies demonstrating the positive association between adherence to MD and semen quality in terms of sperm count, motility and concentration [80, 90–92]. Similarly, evidence from RCTs conducted on healthy young men demonstrated a positive effect on the semen quality of MD in comparison to a low-fat diet [93] or habitual diet [94]. Such beneficial effects are mainly due to both anti-inflammatory and antioxidant potential exerted by bioactive compounds contained in foods characterizing the MD [13, 95, 96]. Among these, dietary lipids play a prominent role.

In general, MD stands out for the low intake of saturated fatty acids (SFAs) and trans fatty acids, whose negative effects on sperm quality have been reported in animal-based studies [97–99]. On the contrary, MD ensures adequate intakes of both mono and polyunsaturated fatty acid (MUFA) and PUFA sources [100], able to modify the lipid composition of spermatozoa membranes, improving their energy metabolism [98]. More specifically, MUFA (mainly contained in extra virgin olive oil) exerts a marked antioxidant effect reducing the levels of ROS and restoring mitochondrial function [98]. Reduction of ROS levels is a key target since these oxidants can damage sperm membranes and alter DNA [101], causing epigenetic modifications responsible for the development, among the others, of cancer and infertility [102]. Interestingly, a higher MUFA/SFA ratio is positively associated with sperm concentration and total count, as reported in a cross-sectional analysis on men of sub-fertile couples [103].

The antioxidant activity is also exerted by PUFA n-3, whose action in increasing the aconitase/fumarate activity ratio has been reported [98]. These are two Krebs cycle key enzymes, whose activity ratio is recognized as an indicator of mitochondrial ROS production [104]. The antioxidant potential of PUFA (mainly contained in fish and nuts) is accompanied by their anti-inflammatory activity, as precursors of eicosanoids. Among PUFA, however, n-3 are precursors of anti-inflammatory eicosanoids (such as prostaglandins, thromboxanes, leukotrienes, and resolvins) [105], suggesting the importance to increase the intake of n-3 or increase the n-3/n-6 ratio, as ensured by following MD. In this sense, higher serum n-6/n-3 ratio have been found in infertile males than in fertile ones [106]. In addition, clinical evidence reported that the beneficial effect on male fertility of n-3 sources is weaker than the negative effect of higher consumption of n-6 sources [103]. This can be explained by the fact that testis cells are more able to convert C18 and C20 PUFA n-3 to C22 PUFA n-3 than to convert C18 and C20 PUFA n-3 to C22 PUFA n-3 [107].

Equally remarkable, is the ability of PUFA, in particular n-3, to modulate the activity of enzymes involved in spermatozoa energy pathways, including lactate dehydrogenase-C4 (LDH-C4) [98]. LDH-C4, catalyzing the pyruvate to lactate conversion (with consequent oxidization of NADH to NAD^+^), plays a pivotal role in sperm energy metabolism, allowing the progression of both glycolysis and oxidative phosphorylation [108, 109].

Data from an RCT on healthy men following a Western diet demonstrated that the inclusion of 60 g of a mixture of nuts (walnuts, almonds and hazelnuts) daily for 14 weeks resulted in significant improvements in sperm vitality, total motility, progressive motility, total count, and morphology. Such results were accompanied by a significant reduction of sperm DNA fragmentation, which can explain the observed improvements in sperm quality. Since the diet followed by the intervention group remained the same, except for the addition of nuts, it can be speculated that the beneficial effects observed might be attributed to the increased intake of PUFA, in particular, increased intake of n-3 and n-3/n-6 ratio [110].

The quality of lipids consumed following a Mediterranean-style dietary pattern is ensured by the choice of foods characterizing MD. For example, MD suggests the consumption of low-fat dairy products. Certainly, this suggestion aims to control the intake of cholesterol and SFA, but also it allows the consumption of foods that exert, among others, beneficial effects on male fertility, in terms of sperm vitality and concentration [111]. The consumption of low-fat milk, indeed, is associated with increased levels of insulin-like growth factor-1 (IGF-1) and insulin [112], both required by spermatogenesis to bind and activate the Leyding cell insulin receptors [113]. The consumption of low-fat dairy product typical of MD, thus, on one hand, contribute to reducing the intake of SFA and cholesterol, but on the other provides food alternatives with beneficial effects on fertility. Noteworthy, a not recent case–control study reported a significant association between high intake of SFA and cholesterol and increased risk of testicular cancer development [114].

The consumption of dairy products gained the attention of scientific opinion also for its potential effect in increasing the risk of testicular cancer development. This concern arose from the fact that such products may contain pesticides and veterinary drugs [115–117] acting as endocrine disruptors and female sex hormones [118], which may increase the risk of this cancer development. However, it is not clear whether the absorbed amount of these substances may be such that they effectively increase the risk. Also, there is no strong evidence of the association between high dairy product consumption and testicular cancer risk, and results are conflicting [119] However, MD suggests moderate consumption of such products [100] thus, in absence of evidence disproving it, this suggestion cannot be considered detrimental.

As well recognized, MD is also characterized by a high intake of fruit, vegetable, legumes and whole cereals [100], with positive effects on the control of inflammatory and oxidative status [95], mainly attributable to vitamins and antioxidants contained. Among the first ones, vitamins C and E emerge for their antioxidant activity [13], resulting in improving the overall sperm quality [120–122]. In particular, vitamin E (mainly contained in nuts, oils and seeds) protects spermatozoa membranes against ROS-caused lipid peroxidation, reducing the risk of structural and functional damage [123].

In addition to vitamins, several foods typically included in the Mediterranean dietary pattern are rich sources of bioactive compounds with antioxidant effects, mainly polyphenols. This, in addition to other features, allows attributing a nutraceutical potential to the MD [124].

In general, polyphenols are able to contrast inflammation and oxidative stress via the down-regulation of specific signaling pathways, including NF-κB and MAPK pathways [95]. Besides this principal activity (that per se elucidates a beneficial effect on male fertility of this class of bioactive compounds and by extension of MD), also relevant is their ability to target mitochondria, modulating their metabolism, as well as ROS homeostasis [13]. Although this can be interpreted as general activity, it is strictly linked to male fertility, since various mitochondrial parameters (i.e. respiratory activity, structure integrity, production of ROS and membrane potential) are related to sperm quality [109, 125, 126]. In this sense, the most important aspect refers to a peculiar biphasic activity of polyphenols, also defined as hormetic effect [13], where such molecules may target sperm mitochondria exerting positive or negative effects on their function, depending on their concentrations [127]. Specifically, *ex-vivo* experiments confirmed that polyphenols at low concentrations (0.1 nM) are able to stimulate the mitochondrial respiration active state, thus increasing the efficiency of this process. On the contrary, at higher concentrations (starting from 10 nM) this effect is lost, and the efficiency of mitochondrial respiration decreases [127]. This biphasic effect is due to the peculiar chemistry of polyphenols, i.e., quercetin. On one hand, they are able to interact directly with mitochondrial membranes [128] and complexes of the electron transport chain [129]. On the other hand, at high concentrations, they interfere with both lipid bilayer and membrane protein altering membrane electric properties [130]. These data are of relevant importance, highlighting the line between diet and supplementation. Digestion processes, indeed, can affect both the chemistry and bioactivity of polyphenols, varying their bioaccessibility and bioavailability [131]. In this context, as reported, the plasma concentration of polyphenols in the European population is around 10 nM [132]. Considering the hormetic effects of polyphenols, thus, the low absorption rate of polyphenols from foods (and not from supplements) may be considered a strength, at least referring to their effect on sperm mitochondria.

Another relevant aspect related to some polyphenols refers to their potential estrogen-like activity, due to their chemical structures, resulting in the interference with the activity of enzymes involved in the biosynthesis and degradation of steroids, acting as multi-functional endocrine disruptors [133]. Reducing the intake of sources of such polyphenols (also known as phytoestrogens) would be recommended in order to avoid potential negative effects on normal sex hormone balances.

In this sense, another advantage of MD is related to the regular consumption of fiber, able to reduce the circulating levels of estrogens or their metabolites [134], whose negative impact on normal semen production is clear [135]. Specifically, fiber may affect the enterohepatic reabsorption of estrogens excreted in bile directly or indirectly by reducing the activity of microbial β-glucuronidase [134].

Overall, this evidence demonstrates the relevant potential of MD in improving sperm quality, thus exerting a beneficial effect on male fertility. Although in vitro and animal-based studies elucidated the effects of Mediterranean food-derived bioactive compounds and their mechanisms, it should be noted that the majority of the available evidence on human are from observational studies. This is almost understandable since interventions with unhealthy dietary patterns are not ethical. However, only an association can be observed with these studies, but eventual cause-effect relationships cannot be proven [62]. Further prospective studies and RCT are needed to confirm the effect of MD or Mediterranean food consumption on male fertility and testis cancer.

### Female gonadal axis and Mediterranean diet

#### Point of view of the endocrinologist

Infertility is defined as the failure to establish a clinical pregnancy after 12 months of regular, unprotected sexual intercourse. According to Borght and Wyns, infertility affects between 8 and 12% of couples of reproductive ages worldwide [136].

Female infertility contributes to 35% of overall infertility cases [137]. Several factors influence the possibility of spontaneous conception including the age of the female partner and infertility related to endocrine dysfunction [136]. In fact, regarding age, the possibility of achieving a spontaneous pregnancy decreases with age before conception: it is established that the decline in female fertility begins as early as 25–30 years of age, and the median age of last delivery is 40–41 years in most populations experiencing natural fertility [136].

Healthy nutrition and lifestyle play a major role on reproductive function in both women and men, representing a pivotal factor for all physiological processes related to fertility, such as ovulation, fertilization, implantation, placental growth, and nutrients supply to the fetus [138].

Importantly, alterations of female gonadal axis are often related to altered eating habits [139], since energy balance and reproduction are strictly regulated in hypothalamic neurons of the arcuate nucleus [140]. Different nutritional regimens have been associated with better fertility outcomes, even if the mechanistic links between diet and fertility is still a matter of debate [95]. Inflammation represents a key detrimental mechanism disrupting female fertility and represents a hallmark of the most common endocrine disorders of female gonadal axis, such as PCOS and endometriosis. The main anti-reproductive effects of inflammation can alter menstrual cyclicity, implantation and fetus growth [141].

ROS play a major role in the development of oocytes, corpus luteum function, embryo and its local environment. Therefore, oxidative stress is able to severely interfere with both natural and assisted fertility, and antioxidant therapies represent a hallmark in the counseling of the infertile couple. The most common endocrine alteration of female gonadal axis disruption is represented by PCOS, which is known to affect between 10 and 15% of the population [142]. The etiology of this syndrome is still controversial and debated: PCOS is the expression of a complex functional alteration of the reproductive system and not the consequence of a specific local ovarian or central defect. Insulin resistance represents a metabolic hallmark of PCOS [143, 144]. Hence, it can be concluded that PCOS is the resultant of different pathogenetic mechanisms. Treatment of PCOS should aim to normalize menstrual cycles, restore ovulation and thus fertility, reduce the clinical signs of hyperandrogenism and halt the tendency for progressive aggravation of the disease [145].

When the goal of the treatment is the seek for pregnancy, therapy should aim to correct anovulation, which is responsible for infertility. Weight reduction should be the first recommendation in patients with PCOS and with obesity: obesity, low-grade chronic inflammatory status, and insulin-resistance often coexist in PCOS [15]. In fact, weight loss of at least 5% from baseline weight can improve or even normalize ovarian function [146]. Weight loss reduces insulin, sex hormone-binding globulin (SHBG), and estrogen levels; moreover, excess weight before conception is a major risk factor for fertility outcomes and it is well established that weight loss improves fertility in women with overweight and obesity [137, 147].

#### Point of view of the nutritionist

The role that nutritional components and dietary habits play in modulating the risk of gynecologic diseases, such as PCOS, endometriosis, several gynecologic malignancies and infertility has long been debated [148]. According to Gaskins and Chavarro, identifying modifiable lifestyle factors, such as diet, that influence human fertility is of major clinical and public health significance [149]. Diet exerts an important effect on female fertility [150]. Unquestionably, among all the dietary styles that have been investigated, MD has a beneficial impact on female fertility [151]. Moreover, according to a review by Korre and colleagues, in which data from observational, longitudinal, and RCTs were examined, it has been demonstrated that Mediterranean-type diets can improve BMI and body weight, reduce the incidence of T2DM and metabolic syndrome risk factors, decrease cardiovascular morbidity and coronary heart disease mortality, and decrease all-cause mortality [152].

Additionally, as mentioned before, it is important to emphasize how crucial it is to maintain body weight and fat mass percentage in a normal range to improve fertility outcomes. In fact, it is well documented that obesity decreases natural fertility among men and women as well as pregnancy chances after conventional infertility and ART based treatments. Moreover, pregnancy complications are increased in women with overweight, obesity and PCOS [153]. PCOS is a very common endocrine disorder in women of reproductive age [143]. Although there is no cure for PCOS, first-line management includes conservative treatment through lifestyle interventions that emphasize weight loss and dietary modifications, with the goal of improving insulin sensitivity [148, 154].

Therefore, it is still debated how MD can have a positive impact on female fertility. A cohort study conducted by Chavarro and colleagues on 17.544 women who were planning a pregnancy or became pregnant during the study, an association was found between adherence to the pro-fertility diet (similar to MD) and a lower risk of infertility caused by ovulation disorders [155]. The pro-fertility diet was characterized by lower consumption of trans fatty acids and higher consumption of MUFAs and plant-based protein, as well as decreased consumption of animal protein, low glycemic index foods, and high-fiber foods. Women who followed the pro-fertility diet consumed more nonheme iron and more frequently, that is, at least 3 times a week, took multivitamins, particularly B vitamins (e.g., folic acid) [155].

In a review article by Gaskins and Chavarro, two studies were reported that revealed that adhering to MD resulted in a higher probability of engaging in both physiologic and post-IVF pregnancy [149]. The first case–control study on 485 women of the Seguimiento Universidad de Navarra project, found that women with the highest adherence to a Mediterranean-style diet, characterized by a higher intake of vegetables, fruits, fish, poultry, low-fat dairy products, and olive oil, were 0.56 (95% CI 0.35–0.95) more likely to seek medical attention for pregnancy difficulties [156]. In the second study, involving one 161 couples undergoing IVF/intracytoplasmic sperm injection (ICSI) treatment, it was observed that a higher adherence to MD before treatment was associated with a higher probability of pregnancy after IVF [157].

Lastly, in order to give a specific focus on the effects of MD and PCOS, in a study by Barrea and colleagues, adherence to MD and association with clinical severity of PCOS was assessed in a cohort of 112 treatment-naïve women with PCOS compared with a control group of 112 healthy women matched for age and BMI [15]. It was observed that women with PCOS consumed less extra virgin olive oil, legumes, fish/fish-based foods and nuts than the control group; it was also observed that women with PCOS consumed lower amounts of complex carbohydrates, fiber, MUFA and PUFA n-3 and higher amounts of simple carbohydrates, total fat, SFA and PUFA n-6 than the control group. Therefore, the authors conclude that there is an association between adherence to MD and clinical disease severity in women with PCOS. This association could support a therapeutic role of foods and nutrients from the Mediterranean dietary pattern in the pathogenesis of PCOS, likely involving inflammatory status, insulin resistance and hyperandrogenism [15].

### Neuroendocrine tumors and Mediterranean diet

#### Point of view of the endocrinologist

NETs are tumors, which originate from neuroendocrine system. They generally occur in the digestive and respiratory systems [158]. A strong link between inflammation and neuroendocrine carcinogenesis has been widely reported [158, 159]. Chronic inflammation and pro-inflammatory cytokines contribute to the development and progression of NETs [160, 161]. The host immune system plays an interesting dual function in carcinogenesis, acting as pro- or anti-tumorigenic, depending by the balance of pro- and anti-inflammatory molecules and by the type of tumor-infiltrating immune cells [160, 161]. In the carcinogenesis process the host-mediated anti-tumor activity is inhibited, while the pro-inflammatory mediators are activated and contribute to shape the tumor microenvironment (TME) favouring tumor growth and progression [160].

TME is a compartment that changes during cancer process; it is composed by neoplastic cells, vascular components including blood and lymphatic vessels and proangiogenic factors, extracellular matrix with cancer-associated fibroblasts, infiltrating immune cells and pro-inflammatory and oncogenic molecules [162].

Chronic inflammation favours the activation of macrophages, dendritic cells and neutrophils with release of reactive oxygen species and pro-inflammatory cytokines, including TNF-α, IL-6, IL-8, IL-10, IL-17 and IL-1β and proangiogenic factors [163–165]. Notably, TNF-α, IL-6 and IL-17 are involved in tumor growth and promotion [166]. Pro-inflammatory cytokines activate NF-κB and signal transducers and activator of transcription (STAT3) signaling pathways [150] that are strongly involved in the development and maintenance of TME [167].

Chronic inflammation can favour the hyperplasia and dysplasia of neuroendocrine cells, leading to a neoplastic transformation. With a special regard to GEP-NET, chronic inflammation, but also the alteration of gut microbiota can contribute to carcinogenesis [168, 169]. Chronic atrophic gastritis induces hypergastrinemia that stimulating the proliferation of entero-chromaffin like cells favours gastric NET. In addition, hypochlorhydria induces a change in the gastric microbiota favouring colonization and increasing the risk of gastric malignancy [170]. Chronic atrophic gastritis affects the citric acid cycle, which is involved in gastric carcinogenesis [170].

The relationship between chronic pancreatitis and pancreatic NET has not been clearly established [171]. Capurso et al. reported T2DM and chronic pancreatitis as independent risk factors for pancreatic cancer [172]. However, this finding was not confirmed in other recent studies, that, by contrast, supported T2DM as a risk factor for pancreatic cancer [173]. Inflammatory bowel disease, including ulcerative colitis and Chron’s disease, was associated to an increased risk of small bowel NET [174]. Interestingly, large-scale studies reported that bowel NETs arose far from the area involved in the inflammatory disease, suggesting that they were the result of the effect of proinflammatory cytokines more than local inflammatory disease [175]. An interesting relationship between inflammation and appendiceal NETs has been reported in patients with Chron’s disease [176]. In addition, inflammatory bowel disease was also associated to increased risk of colonic and rectal NETs, causing the destruction of the intestinal epithelial barrier and therefore the release of several proinflammatory molecules. Similarly, to bowel NETs, colonic and rectal NETs developed in colon areas free from inflammatory bowel disease, as a likely result of a prolonged state of inflammation [177].

Finally, we must mention that obesity plays a significant role in NET carcinogenesis [178]. Indeed, obesity, that is associated with hyperplasia of white adipose tissue, in addition to favouring a chronic inflammation, also causes hyperinsulinism, predisposes to T2DM, increases IGF-1 secretion, induces oxidative stress, and impairs leptin and adiponectin secretion [179].

#### Point of view of the nutritionist

There is a correlation between low adherence to MD and cancer, and adherence to MD could influence the aggressiveness of different tumor types (i.e., prostate, breast cancer, and melanoma) [180].

MD may have a positive impact on cancer management by various mechanisms. It inhibits tumor cell growth by affecting hormones and intracellular pathways, and it has anti-inflammatory, anti-oxidative, and anti-aggregating effects. Anti-tumoral effects of MD can be attributed to various food components, such as fish, fruits, vegetables, cereals, olive oil, and legumes. These elements are rich in antioxidants, including vitamins A, C, and E, carotenoids, lycopene, flavonoids, PUFAs, and fibers, which are involved in cellular signaling pathways [180].

In particular, antioxidants scavenge ROS and reduce their production, which are involved in the stimulation of cancer development [181]. Flavonoids inhibit the activation of certain pathways, increase the expression of enzymes involved in the catabolism of carcinogens, and stimulate the AMP-activated protein kinase [182]. Fibers act on the gut microbiota, releasing short-chain fatty acids, which can reduce the expression of cancer-promoting factors [183]. Of note, higher dietary fibers intake was associated with a significant improvement in progression-free survival in 128 patients undergoing immune checkpoint blockade, with the most pronounced benefit observed in patients with sufficient dietary fibers intake and no probiotic use [183]. In this regard, MD is a nutritional pattern naturally characterized by a high fibers intake, provided by whole grains, vegetables, fruit and legumes. PUFAs help reduce chronic low-grade inflammation and the expression of inflammatory cytokines, including IL-6, IL-1β, and TNF-α [184]. Red wine is a source of resveratrol and quercetin, which can modulate the cell cycle, induce apoptosis in cancer cells, and act as an anti-inflammatory [185, 186]. However, the beneficial effects of red wine are controversial. In fact, it should be considered that other distinguishing features of the MD are the richness of virgin olive oil, fruit, cereals and vegetables, which are also good sources of polyphenols and other phytochemicals, and do not present the risks of wine [186].

For all these reasons, MD could be considered a useful model to adopt in patients with NET. However, there is still little scientific evidence on the role of MD in NET. Only one case–control, cross-sectional study, aimed to investigate the nutritional status and adherence to MD in patients with GEP-NETs [187]. A validated 14-item questionnaire called PREvención con DIeta MEDiterránea (PREDIMED) was used to assess adherence to MD. Authors found that patients with more aggressive disease had lower adherence to MD compared to those with G1, localized, and free/stable disease status. Additionally, patients with poor adherence to MD had a higher incidence of metastases and progressive disease. The study suggests that MD may be an important tool for preventing cancer aggressiveness in patients with neuroendocrine tumors, although further studies are necessary to confirm this finding [187].

In conclusion, MD has been found to have a positive impact on cancer management through various mechanisms, including inhibition of tumor cell growth, anti-inflammatory, anti-oxidative, and anti-aggregating effects. The food components of MD, such as antioxidants, flavonoids, fibers, and PUFAs, are involved in cellular signaling pathways that may help reduce cancer-promoting factors. While MD could be considered a useful model in patients with neuroendocrine tumors, there is still little scientific evidence on its role in this specific type of cancer. Therefore, future well-designed studies are needed to better explore the promising role of MD in neuroendocrine tumors.

## Conclusion

Research has suggested that MD may have the potential to prevent and manage endocrine disorders, such as thyroid disorders, gonadal disorders, and neuroendocrine tumors. Studies have indicated that MD could potentially reduce the risk of nodular thyroid disease and thyroid cancer, enhance reproductive health in both men and women, and have a beneficial effect in the management of neuroendocrine tumors. The beneficial effects of MD could be attributed to its anti-inflammatory and antioxidant properties and its high levels of phytochemicals. It is important for endocrinologists and nutritionists to collaborate to optimize patient care and improve the management of endocrine disorders. While further research is necessary, current evidence supports the potential protective effects of MD against endocrine disorders and incorporating it into dietary recommendations could be advantageous.
